# Zidovudine enhances activity of carbapenems against NDM-1-producing Enterobacteriaceae

**DOI:** 10.1093/jac/dkab184

**Published:** 2021-06-13

**Authors:** Yanmin Hu, Anthony Coates

**Affiliations:** 1 Institute for Infection and Immunity, St George’s University of London, London, UK; 2 Helperby Therapeutics Group plc, London, UK

## Abstract

**Objectives:**

To investigate the efficacy of zidovudine in combination with carbapenems against NDM-1-producing Enterobacteriaceae.

**Methods:**

MICs were determined using the broth microdilution method. The combinatory effects of zidovudine and carbapenems were examined using the chequerboard method and time–kill analysis.

**Results:**

We found that the NDM-1-producing strains were resistant to all carbapenems tested. FIC index from chequerboard assay demonstrated that zidovudine synergized with carbapenems against all the NDM-1 strains. Time–kill analysis demonstrated significant synergistic activity when a low level of zidovudine was combined with meropenem.

**Conclusions:**

Zidovudine in combination with carbapenems produced synergistic activity against NDM-1 Enterobacteriaceae strains *in vitro*.

## Introduction

Carbapenemase-producing Enterobacteriaceae (CPE) strains are associated with a reported mortality rate of up to 40%–50% in those patients who are infected.^1^ The rapid emergence of CPE, which are often resistant to many other antibiotics, has left the world with colistin as the last-resort treatment option, although colistin is associated with both nephrotoxic and neurotoxic side effects.^2^ Therefore, it is crucial to boost the effectiveness of carbapenems against CPE.

Previously, we showed that zidovudine (3′-azido-3′-deoxythymidine, previously known as azidothymidine) boosted the activity of colistin both *in vitro* and *in vivo* against multiple strains of resistant Enterobacteriaceae that produced ESBLs or NDM-1 or carried the mobilized colistin resistance (*mcr*) gene.^3^ Zidovudine is an antiretroviral drug that is used in combination with other antivirals to prevent and treat HIV/AIDS. It inhibits viral reverse transcriptase and was the first effective treatment for HIV/AIDS.^4^ Synergy between zidovudine and other non-polymyxin antibiotics has not been published previously.

In this study, we tested, for the first time to the best of our knowledge, the *in vitro* activities of zidovudine in combination with carbapenems against NDM-1-producing Enterobacteriaceae.

## Materials and methods

### Bacterial strains and growth conditions

The bacterial strains harbouring a *bla*_NDM_ plasmid were ATCC BAA-2469 (*Escherichia coli*), ATCC BAA-2470 (*Klebsiella pneumoniae*), ATCC BAA-2471 (*E. coli*), BAA-2472 (*K. pneumoniae*), ATCC BAA-2473 (*K. pneumoniae*) and NCTC 13443 (*K. pneumoniae*). The bacterial isolates were grown in nutrient broth (Oxoid, UK), on tryptone soy agar (Fluka, UK) or CHROMagar Orientation plates (BD, UK). Zidovudine and carbapenems were obtained from Sigma–Aldrich, UK.

### Susceptibility tests of carbapenems and zidovudine

MICs of carbapenems and zidovudine were determined using the broth microdilution method, in accordance with CLSI guidelines.^5^ MIC testing was performed using a 96-well microtitre plate (Fisher Scientific, UK) as described previously.^3^

### Chequerboard assays to determine combination effects of zidovudine with carbapenems

Combinations of zidovudine and carbapenems were prepared using 96-well microtitre plates with drug concentrations starting 2-fold higher than their MIC values and then serially diluted in a 2-fold manner as described previously.^3^ The combinatory effects were determined by calculating the FIC index (FICI) of the combination as follows: FICI = (MIC of drug A, tested in combination)/(MIC of drug A, tested alone) + (MIC of drug B, tested in combination)/(MIC of drug B, tested alone). Synergy was defined as an FICI ≤ 0.5; no interaction was identified with an FICI >0.5 but ≤4; and antagonism was identified if the FICI was >4.^6^ For all the wells of the microtitre plates that corresponded to an MIC (no visible growth and adjacent to wells with growth—isoeffective combinations), the sum of the FICs was calculated for each well using the above equation. The minimum FIC (ΣFIC_min_) and the maximum FIC (ΣFIC_max_) for all the isoeffective combinations were recorded in order to capture the activities of drug–drug interactions and to represent the pharmacodynamic interactions of the combinations.^7^

### Time–kill analysis of antibiotics alone and in combination with zidovudine against log-phase bacteria

A range of different concentrations of antibiotics and zidovudine were prepared using 2-fold serial dilutions and were added, alone or in combination, to a log-phase bacterial culture containing between 1 × 10^7^ and 5 × 10^7^ cfu/mL, and incubated at 37°C. Viability, expressed as log cfu/mL, was determined at 0, 2, 4, 8 and 24 h of incubation by plating out 100 μL of serial dilutions of the cultures onto tryptone soy agar plates. The colonies on the agar plates were counted using an aCOLyte colony counter (Synbiosis) and analysed with the counter’s software. Synergistic activity was confirmed as a ≥2 log_10_ decrease in cfu counts at 24 h of the combination compared with the antibiotic alone, in addition to a ≥2 log_10_ decrease compared with the 0 h count.^8^

## Results and discussion

The MICs of meropenem, imipenem, doripenem, ertapenem, biapenem and zidovudine were determined for the six NDM-1 strains. As seen in Table S1, available as Supplementary data at *JAC* Online, compared with the antibiotic breakpoints,^9^ resistance to all the carbapenems was found in all strains, although breakpoints for biapenem are not available. Zidovudine MIC was 0.5 or 1 mg/L. Carbapenems are the most effective β-lactams against Gram-negative bacteria containing most β-lactamases including penicillinases and ESBLs. Carbapenems are considered to be the most reliable last-resort treatment for bacterial infections. Here we showed that the NDM-1 strains rendered the last-resort antibiotics ineffective. Therefore, it is important to rejuvenate the activities of carbapenems to bring the most effective drugs back to the patient bedside.

The effects of combining zidovudine with meropenem, imipenem, doripenem, ertapenem and biapenem were determined using chequerboard assays for the six NDM-1 strains. As shown in Table 1, ΣFIC_min_ and ΣFIC_max_ were significantly low, showing the presence of both synergy and no interaction in the combinations of zidovudine with the five carbapenems at different drug concentrations (Table S2) against all six strains tested. No antagonism was observed. We also showed significant reduction (at least 4-fold) in MICs of the carbapenems after combination with zidovudine (Table S2).

**Table 1.** Combination of zidovudine with different carbapenems against NDM-1-producing *E. coli* and *K. pneumoniae*

Bacterial strains	ΣFIC	FICI for ZDV combined with:				
		meropenem	imipenem	doripenem	ertapenem	biapenem
BAA-2469	ΣFIC_min_	0.27	0.19	0.38	0.25	0.16
	ΣFIC_max_	0.63	0.53	0.56	0.56	0.53
BAA-2470	ΣFIC_min_	0.19	0.31	0.19	0.13	0.19
	ΣFIC_max_	0.56	0.56	0.56	0.53	0.53
BAA-2471	ΣFIC_min_	0.13	0.38	0.31	0.25	0.25
	ΣFIC_max_	0.56	0.56	0.56	0.56	0.56
BAA-2472	ΣFIC_min_	0.50	0.31	0.38	0.50	0.25
	ΣFIC_max_	0.63	0.56	0.56	0.56	0.56
BAA-2473	ΣFIC_min_	0.25	0.50	0.38	0.25	0.25
	ΣFIC_max_	0.63	0.63	0.63	0.56	0.53
NCTC 13443	ΣFIC_min_	0.38	0.31	0.38	0.50	0.16
	ΣFIC_max_	0.63	0.56	0.56	0.63	0.53

ZDV, zidovudine.

**Figure 1.**
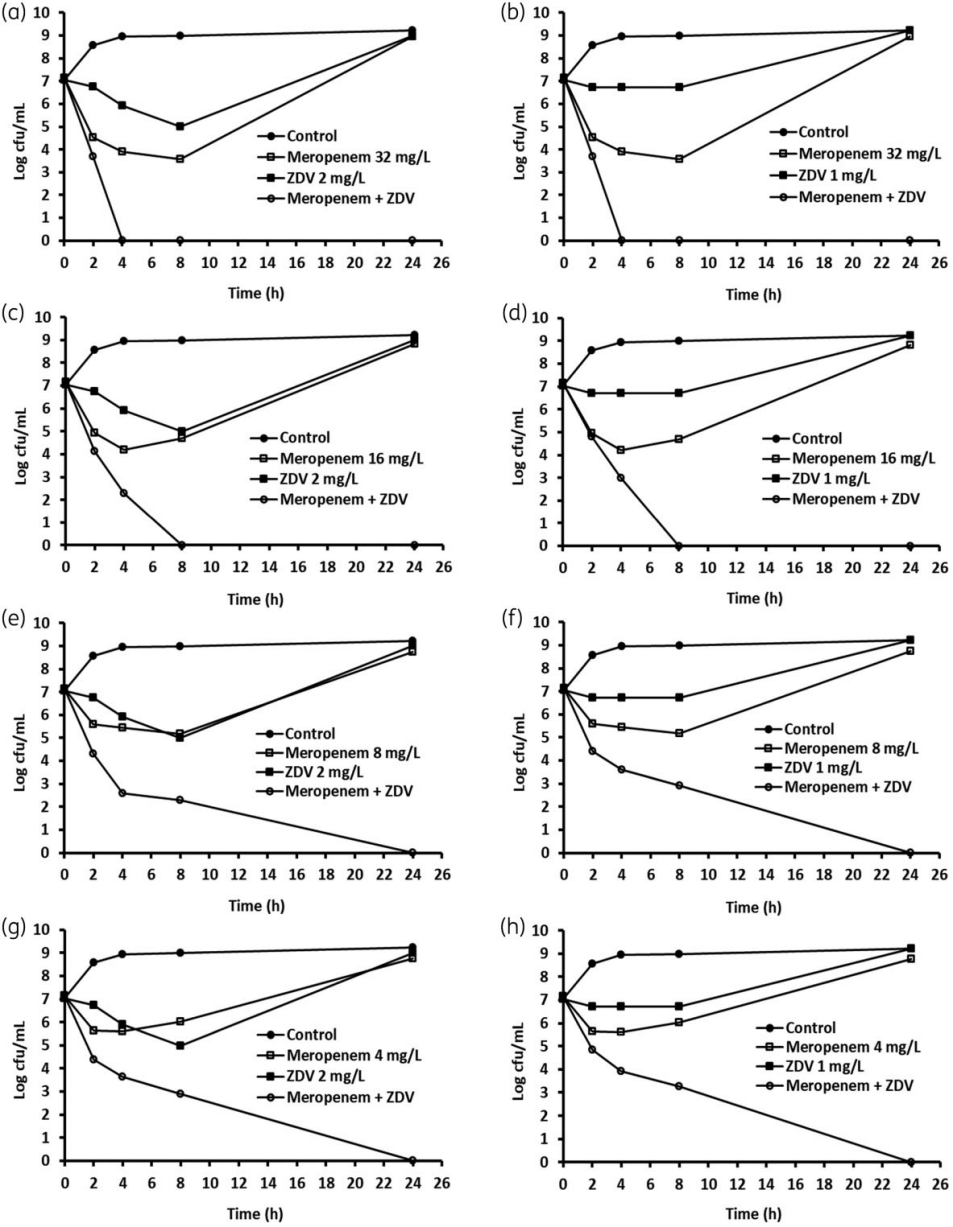
Time–kill analysis showing the effects of zidovudine (ZDV) in combination with meropenem against NDM-1-producing *K. pneumoniae* BAA-2472. ZDV and meropenem alone or in combination were added to the log-phase cultures and cfu counts were carried out at different timepoints.

The bactericidal activity of the synergistic combination of zidovudine and meropenem was determined using time–kill assays for all the NDM-1 *E. coli* and *K. pneumoniae* strains. We used zidovudine concentrations of 1 or 2 mg/L to combine with four different concentrations of meropenem, starting at 2-fold or at the MIC level. As shown in Figure 1, meropenem at 32 (MIC) and 16 mg/L gave rise to a 3 log reduction in cfu counts at 4 h and regrowth was seen after 8 and 4 h, respectively; meropenem at 8 mg/L reduced counts by about 2 log cfu at 8 h, followed by regrowth; and meropenem at 4 mg/L reduced counts by about 1.5 log cfu at 2 h, followed by regrowth. Zidovudine at 2 mg/L reduced counts by 2 log cfu at 8 h, followed by bacterial regrowth; and at 1 mg/L inhibited bacterial growth for 8 h, followed by regrowth. However, when meropenem at 32 and 16 mg/L was combined with zidovudine at 2 or 1 mg/L, complete elimination of cfu counts was observed at 4 h (Figure 1a and b) and at 8 h (Figure 1c and d), respectively. The combinations of meropenem at 8 and 4 mg/L with zidovudine at 2 or 1 mg/L completely eliminated the cfu counts at 24 h (Figure 1e–h). Similar synergistic activity was observed for the other NDM-1 strains (data not shown).

We demonstrated that zidovudine synergized with five different carbapenems against NDM-1 strains, which is the most difficult-to-treat resistant type of Enterobacteriaceae (two *E. coli* and four *K. pneumoniae*). We showed that in combination with zidovudine, the MIC of each carbapenem was significantly reduced (Table S2).

It has been reported that after 600 mg oral dosing of zidovudine, *C*_max_ reached 3.5 mg/L in humans.^10^ When zidovudine was given in a single IV dose of 120 mg on the first day, followed by a single oral dose of 200 mg on the second day, the maximum concentration of zidovudine in serum was 1.751 mg/L.^11^ It is not known if the doses used clinically are sufficient to boost the activities of carbapenems to treat bacterial infections in humans.

We used the published blood concentration of zidovudine to combine with meropenem, and we found that zidovudine at 1 or 2 mg/L significantly boosted the activity of this antibiotic against the NDM-1 strains. After observation of the meropenem time–kill curve profile, we noticed that meropenem was initially bactericidal at the MIC level or below against the NDM-1 strains, then regrowth was seen. The early effect of meropenem might be due to lack of carbapenemase activity that was induced after treatment with the drug.^12^ Combination of zidovudine with meropenem significantly increased the activity of meropenem and showed sustained bacterial clearance over the 24 h drug exposure. It is unknown how zidovudine enhances the activities of carbapenems. Further studies are needed to uncover the mode of action of this combination.

Following on from our initial proof-of-principle data, testing the novel synergistic effects between zidovudine and carbapenems is underway, using a panel of CPE isolated from patients with clinically relevant infections. In particular, the therapeutic activity of zidovudine/carbapenem combinations against highly lethal CPE, including NDM-1 producers, is clinically important. The combination therapies will be tested in animal models. This study paves the way for validation of zidovudine/carbapenem combinations in future clinical trials with the aim of bench-to-bedside translation to benefit patients.

## Supplementary Material

dkab184_Supplementary_Data
