# Supplementary material for: Zidovudine enhances activity of carbapenems against NDM-1-producing Enterobacteriaceae
Source: J Antimicrob Chemother. 2021 Jun 13;76(9):2302–5. doi: 10.1093/jac/dkab184 (PMC8654596; doi:10.1093/jac/dkab184)
Supplement: dkab184_Supplementary_Data [file dkab184_supplementary_data.zip › 21-0401-Supplementary data.docx]

**Supplementary data**

Table S1. MIC of carbapenems and AZT against NDM-1 producing *E. coli* and *K. pneumoniae*

|  | MIC (mg/L) | | | | | |
| --- | --- | --- | --- | --- | --- | --- |
| Bacterial strains | Meropenem | Imipenem | Doripenem | Ertapenem | Biapenem | AZT |
| BAA2469 | 32 | 256 | 64 | 128 | 64 | 1 |
| BAA2470 | 16 | 16 | 256 | 64 | 8 | 0.5 |
| BAA2471 | 128 | 256 | 128 | 256 | 8 | 0.5 |
| BAA2472 | 128 | 128 | 256 | 256 | 32 | 1 |
| BAA2473 | 16 | 8 | 16 | 8 | 8 | 1 |
| NCTC13443 | 8 | 8 | 16 | 8 | 128 | 1 |

Breakpoints ^1^ for meropenem, imipenem, doripenem and ertapenem are 2, 4, 2 and 0.5 mg/L, respectively

1. European Committee on Antimicrobial. The European Committee on Antimicrobial Susceptibility Testing. Breakpoint tables for interpretation of MICs and zone diameters. Version 10.0, 2020. http://www.eucast.org. *Break tables Interpret MICs Zo diameters Version 100* 2020.
